# Preventing sexism and sexual harassment in medical schools by using Theater of the Oppressed as an interactive and reflexive tool

**DOI:** 10.1186/s13104-022-06084-2

**Published:** 2022-06-03

**Authors:** Emmanuelle Lüthi, Lauriane Pichonnaz, Joëlle Schwarz, Pascal Morier-Genoud, Caroline Dayer, Ilire Rrustemi, Léa Schilter, Alexandre Berney, Caroline John, Julie Dubois, Pierre-Yves Rodondi, Carole Clair

**Affiliations:** 1 Institute of Family Medicine, Faculty of Science and Medicine, University of Fribourg, Route des Arsenaux 41, 1700 Fribourg, Switzerland; 2 Gender and Medicine Unit, Department of Training, Research and Innovation, Center for Primary Care and Public Health (Unisanté), University of Lausanne, Lausanne, Switzerland; 3 Department of Psychiatry, University of Lausanne, Lausanne, Switzerland; 4 The Theater Company Le Caméléon, Lausanne, Switzerland; 5 General Secretariat of the Department of Education, Youth and Culture of the State of Vaud, Lausanne, Switzerland

**Keywords:** Medical education, Sexual harassment, Prevention, Social medicine, Theater of the Oppressed

## Abstract

**Objectives:**

Among the measures taken to combat sexism and sexual harassment, prevention courses for medical students are one possibility. We aimed to describe the process of implementing a training course on the prevention of sexism and sexual harassment for medical students in two Swiss medical schools by using the Theater of the Oppressed as an interactive and reflexive tool within the course. The purpose of this theater was to give the students the opportunity to express themselves and to collectively look for and discuss ways to combat and escape from oppressive situations.

**Results:**

This collaborative, innovative, and interactive implementation showed that different forms of a training course can be implemented with similar objectives in an adaptable and transferable manner. The interactive and reflexive Theater of the Oppressed was an appropriate option to reach the objectives. Courses were based on identifying and acting on concrete problematic situations by focusing on individual, collective, and institutional resources. Students reported a high level of satisfaction.

## Introduction

The issues of sexism and sexual harassment (SH) are topical. Following the #MeToo movement, most Western institutions are in crisis regarding these issues, including medical and educational institutions [1]. People are calling for change, including institutional change, and fighting against sexism and SH is an integral part of this call.

Although sexism is not a new phenomenon in the medical field, it has become more visible in the last few years. According to a US report from the National Academies of Sciences, Engineering, and Medicine (NASEM) [2], female medical students are 220% more likely to be sexually harassed than are their colleagues from other faculties. This report points out that, among the factors explaining this higher prevalence, is the hierarchical and dependent relationship between faculty members and their trainees. In addition, medical students and their mentors often spend much time alone together (laboratories, field sites, and hospitals). Moreover, medical trainees are frequently integrated in different structures throughout their curriculum, hindering their capacity to identify the structures available to report SH.

In Switzerland, SH is defined by the Swiss federal law on equality between women and men (art. 4) [3]. Concerning sexism, many definitions exist. We relied on the one by Dayer [4]: “Sexism is an ideological system that dichotomizes and ranks sexes by postulating the superiority of the category of men over the category of women. It is a set of beliefs, behaviors, and actions that (re) produces this alleged superiority, directly or indirectly, at the individual, collective, and institutional levels. This system manifests itself in particular through stereotypes, discrimination, and violence based on gender.”

As education on SH is important for prevention, a training course on sexism and SH was implemented in the medical curriculum of two Swiss medical schools (Lausanne and Fribourg) to initiate social change in this field, alongside a larger institutional prevention program against sexism and SH.

Regarding the appropriate educational approach, Christensen [5] underlines that interactive techniques are more efficient than didactic methods. From this perspective, the Theater of the Oppressed [6], as an interactive and reflexive tool involving active student participation, appeared to be an effective means of bringing about social change. Theater of the Oppressed is a method of personal and social transformation and evolution in situations of oppression, first developed in the 1970s by Augusto Boal [6]. The purpose of this method is to give the participants the opportunity to express themselves and to collectively look for and discuss ways to combat and escape from oppressive situations. There are several forms of Theater of the Oppressed, including Forum Theater and Image Theater.

In this article, we aim to describe the process of implementing such a training course from the authors’ experience of setting up the course, along with the results from an external expert evaluation.

## Main text

### Methods

**Fig. 1:**
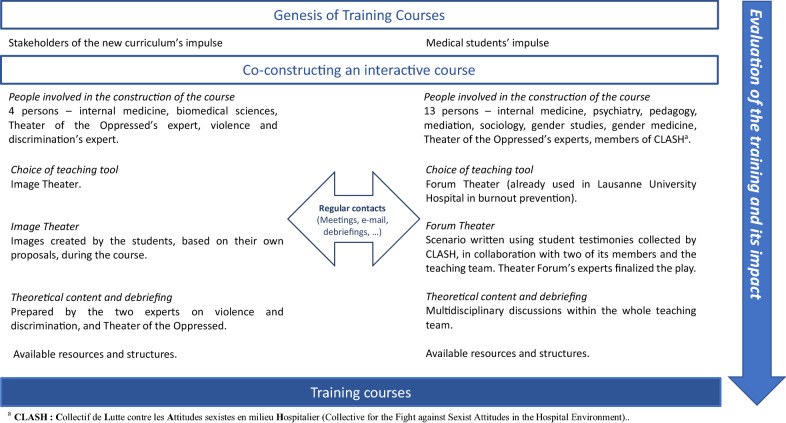
Process of implementation for the course on sexism and sexual harassment prevention for medical students

The Theater of the Oppressed was first considered because of previous favorable experiences with this tool by some members of our teams, and because of the importance of participatory and innovative teaching on the topic of sexism and SH. Depending on the preferences of the stakeholders, two different forms of the Theater of the Oppressed were chosen. Table 1 describes them and Fig. 1 describes the implementation process.

**Table 1:** Description of Forum Theater and Image Theater with examples from each university

Theater Form	Description	Example
Forum Theater in the medical school of Lausanne	In the Forum Theater, the actors play a story that stages problematic situations in which characters are either targets, authors, or bystanders. The actors then replay the playlets and, invited and guided by a facilitator, the audience intervenes and changes the course of the story. When a participant intervenes, he or she can go on stage to replace a character and propose alternative scripts and solutions to respond to the oppression. Participants are considered “spect-actors.”[6] In Lausanne, playlets were tailor-made based on the original testimonies collected by CLASH in order to be as close to reality as possible.	Lucie, a 4th-year medical student, is doing a hospital internship. The first scene shows that she experiences everyday sexism, for example, by a professor who considers her as a woman who is going to 1 day be a mother who will not be able to ensure that she is capable of handling a demanding position in the future. The second scene shows her being harassed by text messages from another professor. The last scene, in the operating room, shows the professor who was texting her and his female professor colleague; he harasses Lucie verbally and physically while his colleague supports him and laughs. The play emphasizes different *forms* of sexual harassment, the potential *author(s*) (hierarchy vs peers), the key role of *bystanders*, and how a *hostile environment* can be harmful for the target.
Image Theater in the medical school of Fribourg	Image Theater invites the participants to re-create oppressive situations by impersonating those situations in the form of a life-sized sculpture. The participants in the sculpture are invited to express how they felt in this situation and can propose changes in the life-sized sculpture to improve the situation. This technique enables participants to show and reflect on how, starting from a concrete proposed situation, creating a different relationship with others, as a spect-actor and a bystander, can improve the well-being of all participants.	In a life-sized sculpture proposed by a student, a medical practitioner is touched on the thigh by a person. Next to her are the 3 monkeys that see no evil, hear no evil, and speak no evil. Students would like to show in this life-sized sculpture that *bystanders* are important in oppression situations. They are allowed to support and help the target in many ways, no matter who the *author* of the oppression is.

*CLASH* Collectif de Lutte contre les Attitudes sexistes en milieu Hospitalier/Collective for the Fight against Sexist Attitudes in the Hospital Environment

The objectives of both courses, implemented in two separate medical schools in Switzerland, were to support students in order to (1) identify situations of sexism and SH as targets, authors, or bystanders and (2) act on problematic situations by using tools, institutional or legal policies, and procedures for reporting SH, as taught during training.

Participants of the course were students in either their 3rd or 4th year of medical study. The goal was to provide this course right before the beginning of hospital internship rotations, which take place in different years, depending on the medical school.

As this article describes the process of implementing a training course, no data were collected or analyzed by the authors. In addition, as there was no collection of personal health-related data, ethical approval and consent to participate was not required by the local ethics committee (Ethics Committee Vaud), in agreement with the Swiss Human Research Ordinance (HRO) [7].

## Results

### Genesis of the course

In reaction to their own experiences during their years of internship, a group of medical students at the medical school of Lausanne sent a non-formal questionnaire about sexism and SH to their peers in 2018. Because of the disclosure of many incidents of serious damage to the integrity of students, they decided to form an association [Collectif de Lutte contre les attitudes sexistes en milieu hospitalier (CLASH)] to fight against sexism in the hospital environment. They presented the survey results to the university hospital management and the Faculty deanship and requested, among other measures, the implementation of a training course on sexism and SH prevention for medical students, which was granted. In the same year at the medical school of Fribourg, a training program for students in their 4th to 6th year of medicine had newly opened. The program directors decided to integrate a course on the prevention of sexism and SH from the start of the curriculum.

### Co-constructing a course

The courses were set up by a multidisciplinary team with different experiences and expertise. Stakeholders from internal medicine, psychiatry, pedagogy, mediation, sociology, gender studies (including prevention of violence and discrimination), and gender medicine were involved, as well as stakeholders from the Theater of the Oppressed and members of CLASH.

Although both medical schools decided to build their own courses, regular contacts were maintained between them during and after the construction of the courses. During construction, regular exchanges and meetings between stakeholders of the two schools were organized to ensure a solid common basis for the courses. In addition, some experts were involved in both courses, which helped in the transmission of experiences. Contacts were maintained after course completion to share experiences, discuss the results of the evaluation, and decide on changes to implement.

### Mixing theory, interaction, and discussion

Courses were designed according to three aspects: theory, interaction, and discussion. The theory included (1) the legal definition of SH, (2) the employer’s duty in the case of SH, (3) the impact of SH on health, and (4) institutional resources. The take-home messages for students were to break the silence, not to trivialize, to act with support, and to avoid victim’s guilt and victim shaming, as well as that it is the duty of the workplace to be non-discriminatory and benevolent. All of this information was also provided to students in writing as a vade mecum. For more details, see Table 2.

**Table 2:** Information from the training courses

Course description	Image Theater (Fribourg)	Forum Theater (Lausanne)
Duration	2.5 h	3.5 h
Group size	40 students	20 students
Year of medical study of participants	4th year	3rd year
Stakeholders’ teaching	1 expert in the prevention of violence and discrimination, specializing in gender studies, as well as issues of sexism and sexual harassment 1 comedian, expert in the prevention of discrimination	2 lecturers^a^ 1 member of the association CLASH^b^ 4 comedians
Structure	Students’ representation of the topic Theoretical and legal clarification (with slide support) Image Theater Synthesis/institutional resources (with slide support)	Introduction Forum Theater Discussion/theoretical input/institutional resources (with written support)
Objectives	Identify situations of sexism and sexual harassment as targets, authors, and bystanders Act in problematic situations by using tools, institutional or legal policies, and procedures for reporting sexual harassment, as taught during training	
Summary of content	Definitions of sexual harassment and sexism at the workplace (legal, social, individual) Impact of sexual harassment on health Gender bias Testing ways to fight against sexual harassment and sexism by using theater to practice Trusted addresses and contact information	
Take-home messages	Breaking the silence No trivializing Acting with support Avoid victim’s guilt, victim shaming It is the duty of the workplace (including in internship placements) to be non-discriminatory and benevolent	

^a^From gender and medicine unit and from liaison Psychiatry Unit

^b^Collectif de Lutte contre les Attitudes Sexistes en milieu Hospitalier/Collective for the fight against sexist attitudes in the hospital environment

### Evaluation of the training

Funded by the Swiss Federal Office for Gender Equality, we mandated an external expert evaluation, which was based on the observation and questionnaires of these two courses, as well as on students’ and stakeholders’ interviews [8]. The goals were to measure the effects of the courses and the achievement of their objectives, as well as to contribute to their optimization. The authors of the expert evaluation concluded that the context justified the implementation of these training courses, that the course objectives were met, and that the interactive form of the Theater of the Oppressed was an appropriate option to reach the objectives. Indeed, the Theater of the Oppressed allowed students to experiment with strategies (verbal and nonverbal) and approaches to transformation in a safe environment and in a collective way, which enhances understanding of discrimination and oppression. Moreover, many students became aware of the importance of the role of the bystander(s). They also recognized male privilege. The students had a very high level of satisfaction and reported a high perception of acquired knowledge related to sexism and SH. The authors of the expert evaluation observed a strong willingness of students to take part in learning about sexism and SH as a societal issue. Nevertheless, some students felt discouraged and alone in facing sexism and SH, with the feeling that the institution that trains them will not change. The authors of the evaluation concluded that the potential of course transferability was strong. Finally, they made some recommendations for future courses, such as making them mandatory for students, providing a reminder later during the curriculum, and delivering the course to teaching staff [8].

## Discussion

In the training contexts in which they perform, students are at risk of sexism and SH, as shown in the scientific literature [2] and through student testimonies. In this article, we aimed to describe two similar initiatives for prevention of sexism and SH to ensure healthy and safe training environments for medical students. Existing studies suggest that training can improve knowledge of SH procedures and lead to a better understanding of the phenomenon [2]. In addition, a reduction in SH is strongly associated with behavioral changes [2], and the techniques used in the Theater of the Oppressed, which involve both the body and the mind in interactive and concrete situations, can facilitate this change. The goal of the training courses was to discuss and identify individual, collective, and institutional resources to fight against sexism and SH. Addressing these topics requires mandatory and repeated teaching delivered by experts with deep knowledge about sexism and SH, who are able to generate an exchange of ideas, because SH, and sexism affect the very functioning of the society in which the students evolve.

Because SH impacts on the psychological, physical, professional, and social well-being of persons [9] and because SH is one of the various expressions of systemic sexism [10], the reflection was performed on three levels: individual (body posture and professional practices), collective (reference group and professional culture), and institutional (managers and communication).

This “three-level” approach is innovative in the sense that (1) it addresses potential situations of SH that could occur within participants’ own institutional settings, whereas these topics are generally debated outside of the institution; (2) it is in line with Swiss guidelines for medical education for professionalism and accountability; and (3) it is co-constructed by a variety of stakeholders.

According to the three focuses of prevention highlighted in report by the NASEM [2] (prevention, protection, accountability), such courses are not sufficient: Trainers must also benefit from this kind of training in order to guarantee balanced prevention [11]. Furthermore, in Switzerland, there is no legal protection against SH in higher education, as the Swiss federal law on equality between women and men covers only salaried employment relationships. As a result, denunciations are rare (especially because of fear of reprisals) and sanctions even rarer, and students who are victims of SH often remain isolated [2]. Therefore, prevention measures have to be implemented in academic institutions. These institutions must take responsibility, bearing in mind that these situations do not, or do not only, result from bad individuals, but are also part of an organizational culture that tolerates situations of sexism [12]. With a strong and clear message of “zero tolerance,” institutions must provide transparent and accessible policies, procedures, and resources to address SH [11] in order to promote cultural change [13].

## Conclusion

It is feasible to implement a training course on the prevention of sexism and SH for medical students by using Theater of the Oppressed. Such courses might help students better identify and react as victims or bystanders when facing situations of sexism and SH. They also have the potential to change behaviors, prevent future sexist behaviors, and contribute to a cultural change. However, such training courses need to be accompanied by other measures, such as making them compulsory for the entire clinical staff.

## Limitations

One limitation of the current study is that this course might be difficult to replicate in other universities, depending on the possibility of integrating new courses in their current medical curriculum and their available financial resources. Furthermore, although the Theater of the Oppressed was chosen to deal with this topic, other tools unknown to us may exist that would be appropriate too. Finally, the course is held only once in the curriculum. Prevention would be more complete if the topic were to be repeated longitudinally throughout the curriculum.

## Data Availability

Data sharing is not applicable to this article as no datasets were generated or analyzed during the current study.

## Abbreviations

CLASH: Collectif de Lutte contre les Attitudes Sexistes en milieu Hospitalier/ Collective for the Fight against Sexist Attitudes in the Hospital Environment
SH: Sexual harassment
